# Are you with me? Probing the human capacity to recognize external/internal attention in others’ faces

**DOI:** 10.1080/13506285.2018.1504845

**Published:** 2018-08-07

**Authors:** Mathias Benedek, David Daxberger, Sonja Annerer-Walcher, Jonathan Smallwood

**Affiliations:** a Institute of Psychology, University of Graz, Graz, Austria; b Department of Psychology, University of York, York, UK

**Keywords:** Internal attention, recognition, facial expression, imagination

## Abstract

Facial cues provide information about affective states and the direction of attention that is important for human social interaction. The present study examined how this capacity extends to judging whether attention is internally or externally directed. Participants evaluated a set of videos and images showing the face of people focused externally on a task, or internally while they performed a task in imagination. We found that participants could identify the focus of attention above chance in videos, and to a lesser degree in static images, but only when the eye region was visible. Self-reports further indicated that participants relied particularly on the eye region in their judgements. Interestingly, people engaged in demanding cognitive tasks were more likely judged to be externally focused independent of the actual focus of attention. These findings demonstrate that humans use information from the face and especially from the eyes of others not only to infer external goals or actions, but also to detect when others focus internally on their own thoughts and feelings.

Looking at the faces of members of the audience while delivering a lecture often leaves the impression that some attendees are absorbed by thoughts unrelated to the topic of the talk. Studies have shown that people do devote significant time to states of internal focus in many different situations (Smallwood & Schooler, 2015) including lectures (Seli, Wammes, Risko, & Smilek, 2016). But is our sense of confidence in our judgments of other people’s focus of attention warranted – can we really tell attentional focus from the face? And if so, what aspects of the face are important? The present study addressed these questions by testing whether participants can accurately identify the focus of another’s attention using facial information, and examined how recognition depends on the type of cognitive task and whether the eyes region is a particularly important discriminate aspect of the face.

We know that humans use facial information to great effect when attempting to understand another’s behaviour. For example, facial expressions provide a wealth of information on affective states, and humans are capable of recognizing many different basic emotions based on a person’s facial features (Ekman, 1993). The eyes are particularly important in inferring information about conspecifics, and we use gaze-related information to efficiently and rapidly infer where in space another individual is attending (Emery, 2000), a process that emerges early in development (Farroni, Johnson, Brockbank, & Simion, 2000), and is critical to social interactions (Itier & Batty, 2009). These studies demonstrate that we efficiently use facial information to understand another person’s actions, but it remains unclear whether this information can be used to detect states in which attention is not directed externally.

Attention is a core cognitive function responsible for the selection and maintenance of focus on relevant information and its focus depends on the type of cognitive task: externally directed cognition (EDC) such as reading and visual search involves attending to external, perceptual cues, whereas internally directed cognition (IDC) such as planning and imagination involves attending internally to thoughts and memory (Dixon, Fox, & Christoff, 2014). Due to limited information processing capacity, EDC and IDC are considered competing mental states (Chun, Golomb, & Turk-Browne, 2011). EDC and IDC are associated with unique neural correlates (Benedek et al., 2016; Dixon et al., 2014) and specific patterns of eye behaviour (Konishi, Brown, Battaglini, & Smallwood, 2017; Reichle, Reineberg, & Schooler, 2010; Walcher, Körner, & Benedek, 2017). Contemporary accounts of attention also acknowledge that cognition varies in the level of goal-directedness and cognitive demand (Christoff, Irving, Fox, Spreng, & Andrews-Hanna, 2016; Smallwood & Schooler, 2015). More demanding tasks usually put higher constraints on thought, making them potentially more predictable.

To understand whether inferences on the focus of attention can be made from observing others’ faces, we produced video recordings of participants while performing cognitive tasks that varied on whether attention has to be directed internally or externally (i.e., EDC vs. IDC tasks). Tasks further varied in the level of cognitive demand as we assumed that recognition ability might depend on how strongly task demands constrain ongoing thought. Additionally, this manipulation enabled us to test whether judgments are consistent across different EDC/IDC tasks or potentially biased towards external or internal states for higher task demands. An independent group of participants then rated the attentional state of these individuals. To determine whether these judgments exploited temporal dynamics of the face, faces were rated as both video, and as static images. Moreover, to examine if the eyes were particularly important in providing a signature of different attentional states we compared these judgements when the whole face was available and when we masked out the eyes images. Before and after this evaluation task, participants also judged their own attention recognition ability, which allowed us to test how self-estimates align with actual performance.

## Methods

### Participants

A sample of 10 participants served as actors for the video recordings used in the attention evaluation task (50% female; mean age 26.5, *SD* = 11.2). Data from additional three participants were recorded but excluded due to slightly lower video quality (i.e., videos that either were over- or underexposed, blurred or pixelated in one of the four experimental conditions) according to a thorough screening of all materials by one experimenter. An independent sample of 108 participants (75% female; mean age = 26.4, *SD* = 9.3) completed the attention evaluation task. Participants were university students recruited via the university’s email list. They received an invitation to perform the task at an online test platform and received partial course credits in exchange for participation. Data from additional 10 participants were excluded because of evidence of careless responding: 5 spent less than 15 min on the attention evaluation task (which is about 2 SD below the median working time), and five showed extreme responses (i.e., maximal confidence ratings) on more than 25% of trials (the total average rate of extreme responses was only 1.2%). The employed sample size ensures that small to medium effect sizes of *d* ≥ 0.30 will be tested with high statistical power (>0.90) in within-subject comparisons and one-sample *t-*tests. All participants gave written informed consent.

### Development of the attention evaluation task

#### Rationale and realization of attention states

We created a stimulus set of short video clips featuring the faces of people engaged in cognition relying on either externally or internally directed attention (i.e., EDC vs. IDC) at two levels of cognitive demand (low vs. high). Cognitive demand was used as additional factor as it allowed us to test whether more demanding tasks are more easily recognized. Low-demand EDC was induced by having participants watch a short video showing a tractor transporting beach chairs and asking them to count the number of journeys made by the tractor. Low-demand IDC was realized by asking participants to imagine themselves to be on this beach and explore the environment. High-demand EDC was induced by asking participants to find solutions to a five-letter anagram presented on a screen. High-demand IDC was realized by having participants solve a five-letter anagram in their mind’s eye, as the stimulus disappeared after 1 s. Each task took about 90 s and was followed-up by task-specific questions including the number of transports (low-demand EDC), a brief description of the envisioned beach scene (low-demand IDC), and the produced anagram solutions (high-demand EDC and IDC). The answers documented that all participants were fully engaged in the instructed tasks. Further support for the validity of the employed experimental manipulation of attention focus comes from previous research demonstrating consistent differences between EDC/IDC conditions with respect to brain activation and eye behaviour (Benedek, Bergner, Könen, Fink, & Neubauer, 2011; Benedek, Schickel, Jauk, Fink, & Neubauer, 2014; Benedek, Stoiser, Walcher, & Körner, 2017; Benedek et al., 2016).

#### Video recording and post-processing

Participants sat in front of a screen (1.4 m distance) that either presented the task stimulus (EDC conditions) or a fixation cross (IDC conditions). They were videotaped (1920 × 1080 px, 30 fps) during task performance by a digital camera (Pentax K50 DSLR) placed right next to the screen. The four tasks were completed in a fixed sequence (EDC-low, IDC-low, EDC-high, IDC-high). After the tasks, participants reported whether their task focus had been highest in the beginning, middle, or end of the 90-s task, which guided the selection of the stimuli from the video recordings for the subsequent study.

**Figure 1.**
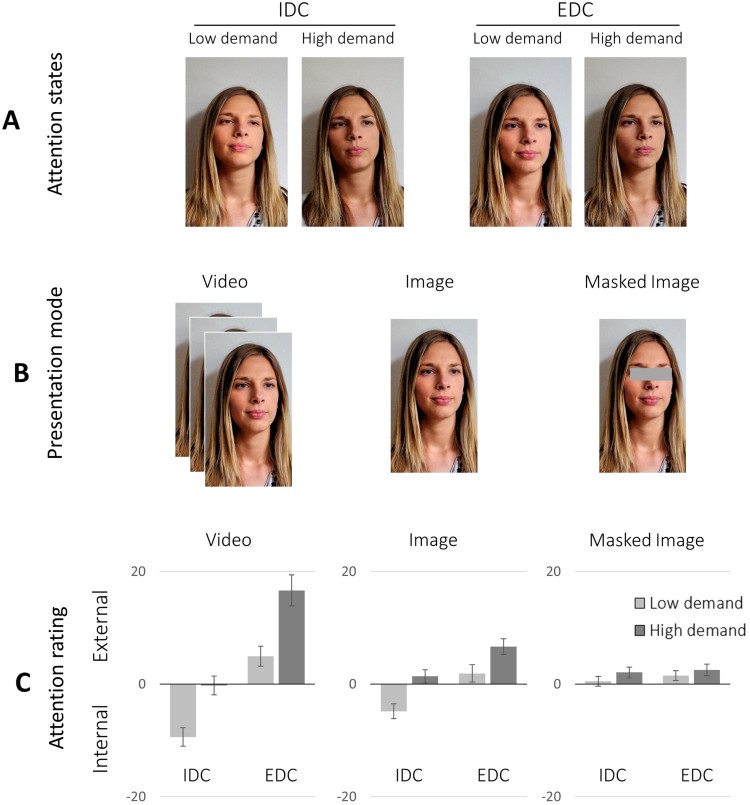
Panels A and B: Example face stimuli representing different attention states and presentation modes. Panel C: Attention recognition performance by attention direction, task demand, and presentation mode (error bars represent 95% confidence intervals). Example face images used with kind permission of the participant.

For an experimental variation of the amount of information from the face stimuli, we created a 5-s video excerpt (video condition), a static face image (image condition), and an image with masked eyes (masked image condition) from each video. Focusing on the video segment with highest reported task focus, we first checked the middle video frame: If it showed the participant with eyes open, it was used as centre frame of the 5-s video excerpt and as image for the static image conditions; if not, this procedure was applied to a recording position 10 s earlier or later. The masked image was produced by occluding the eye and lids with a grey bar. The final attention evaluation task thus consisted of 120 stimuli: 10 people engaged in four attentional states (EDC/IDC with low/high cognitive demand; see Figure 1(A)), presented at three levels of information (video; image; masked image; see Figure 1(B)).

### Procedure

The attention evaluation task was realized with SoSci Survey (Leiner, 2014) and presented online via www.soscisurvey.de. The task started with three example items and participants judged their own ability to differentiate between EDC and IDC on the basis of such facial displays using a continuous slider scale ranging from “very bad” to “very good” (0–100). Then, all 120 face items were presented in randomized order and participants evaluated each stimulus using a continuous slider scale ranging from “attention is clearly internally directed” (−50) to “attention is clearly externally directed” (50). At the end, participants judged their own discernment ability again and were given the opportunity to describe what cues they had relied on in their judgements. The whole task took about half an hour. The study procedure was approved by the local university’s ethics committee.

### Analysis strategy

Attention recognition ability was examined in several complementary steps. To understand whether people can reliably distinguish between external and internal focus of attention in others, we computed hit rates defined as the relative number of correctly identified external and internal mental states (i.e., negative and positive ratings for external and internal targets, respectively). Hit rates with confidence intervals higher than 50% indicate recognition ability above chance level. Hit rates were computed for each presentation mode (i.e., video, image, masked image), to test how this ability depends on the level of accessible information.

Next, we analyzed how attention recognition depends on the type of mental state (i.e., EDC/IDC with high/low cognitive demand) in each presentation mode. To this end, we computed average attention ratings for each condition and tested whether ratings differ significantly from zero (i.e., external states showing negative attention ratings, and internal states showing positive ratings, with confidence intervals excluding zero). Looking at the ratings rather than correct responses provides additional information about the certainty of judgements. Moreover, we formally analyzed differences in attention judgements between experimental conditions by means of ANOVA.

Finally, we analyzed individual justifications to obtain further evidence on what facial cues were used to infer the target’s focus of attention. Participants’ individual justifications were coded as either referring to cues in the eye region, forehead, or lower face (Ekman, 1977) and frequencies were compared across presentation modes by means of a chi-squared test. De-identified performance data along with analysis scripts are available at Open Science Framework (doi: 10.17605/OSF.IO/VGA53). Image and video stimuli have not been made available in order to protect the rights of the displayed persons.

## Results

### Attention recognition by level of information

The direction of other people’s attention can be recognized above chance level from short video sequences (Hit rate = 62.04%, *CI*_95_ = [60.30, 63.77]; *d* = 1.32) and from static images of faces (Hit rate = 55.79%, *CI*_95_ = [54.47, 57.11]; *d* = 0.84); however, judgments become unreliable when images do not include the eye region (Hit rate = 51.04%, *CI*_95_ = [49.77, 52.31]; *d* = 0.16). An ANOVA further revealed that the three presentation conditions differed significantly from each other (*F*[1.78,190.02] = 62.06, *p* < .001, $\eta_{p}^{2}$ = 0.37), with video stimuli yielding higher performance than static images, and static images yielding higher performance than masked images (all *p*s < .001).^1^ These findings indicate that consideration of the eye region improves the evaluation of other people’s attentional focus and that dynamic information in video sequences contributes to better discrimination.

### Attention recognition by mental activity

Next, we explored whether the identification of an individual’s attention depends on the displayed mental activities (IDC/EDC with low/high cognitive demand; see Figure 1(C)). EDC was correctly recognized in videos and unmasked images for both undemanding and demanding EDC (i.e., positive ratings, with confidence intervals excluding zero). IDC was identified correctly in videos and unmasked images for targets showing IDC with low cognitive demand (i.e., negative ratings, with confidence intervals excluding zero), whereas demanding IDC was not reliably recognized as internal focus of attention. The masked face images yielded no reliable recognition.

We tested the effects of attention direction and task demand by means of 2 × 2 ANOVA with factors *attention* (EDC vs. IDC) and *task demand* (low vs. high) for each presentation mode.^2^ In video excerpts, people engaged in EDC were judged as more externally focused than during IDC (*attention*: *F*[1,107] = 177.46; *p* < .001, $\eta_{p}^{2}$ = 0.62). Interestingly, faces depicting demanding mental tasks were also judged more externally focused (*task demand*: *F*[1,107] = 126.22; *p* < .001, $\eta_{p}^{2}$ = 0.54), independent of the actual direction of attention (attention * *task demand*: (*F*[1,107] = 1.87; *p* = .18, $\eta_{p}^{2}$ = 0.02). The same result pattern was observed for unmasked face images (*attention*: *F*[1,107] = 89.39; *p* < .001, $\eta_{p}^{2}$ = 0.46; *task demand*: *F*[1,107] = 88.78; *p* < .001, $\eta_{p}^{2}$ = 0.45; *attention* * *task demand*: *F*[1,107] = 1.79; *p* = .18, $\eta_{p}^{2}$ = 0.02). Faces with masked eyes yielded no reliable recognition of attention direction (*p*s > .05); yet, more demanding cognitive states were still judged more often to reflect external attention (*F*[1,107] = 8.42; *p* = .003, $\eta_{p}^{2}$ = 0.08). Taken together, people engaged in demanding internal or external mental tasks are more likely viewed as externally focused, which improves recognition rates for EDC but undermines proper recognition of demanding IDC.

### Justifications

In the video condition, judgements mostly relied on cues from the eye region (91%), but much less on the forehead (9%) or lower face (9%) regions (*χ* ^2^[2] = 42.43, *p* < .001). For example, participants commonly mentioned to identify an external focus of attention by a different pattern and speed of eye movements. For face image displays, participants again strongly focused on the eye region (70%) in order to assess gaze direction or signs of “empty gaze,” but hardly reported to consider the forehead (9%) or lower face (13%) regions (*χ* ^2^[2] = 24.21, *p* < .001). In the masked eyes condition, participants obviously never considered the eye region (0%) but occasionally referred to the forehead (22%) and lower head (28%) to justify their judgements (*χ* ^2^[2] = 5.49, *p* = .06).

### Self-reported ability

Self-reported ability to recognize the attentional focus in others was initially quite high (*M* = 70.46, *CI*_95_ = [67.46, 73.47]), but uncorrelated with subsequent performance in all presentations modes (*p*s > .35). After completing the attention evaluation task, self-perceived ability was significantly attenuated (*M* = 57.95, *CI*_95_ = [53.35, 62.56]; *t*[107] = 6.33, *p* < .001, *d* = 0.59), and now self-assessments correlated with judgment accuracy in the video condition (*r* = 0.22, *p* = .03; for image and masked image conditions, *p*s > .70), reflecting an increase by trend (*z* = 1.35, *p* = .09). These findings suggest that people judged their attention recognition ability more adequately after the attention evaluation task.

## Discussion

Our study revealed that humans can recognize the focus of others’ attention using information contained in their facial expressions. The eye region was particularly informative for judgments of an individual’s attentional focus, since accuracy was at chance when no information from the eye region was available. Previous research has established that we use gaze information to make inferences regarding a person’s external goals (Langton, Watt, & Bruce, 2000) as well as more subtle inferences important for social cognition (Itier & Batty, 2009). Our findings extend these observations by showing humans can identify another individual’s internal state. Judgement accuracy increased when facial expressions were presented in a dynamic rather than static manner, suggesting that people may be using subtle changes in cues, such as eye movements, that may reflect the processing of external information (Walcher et al., 2017). This notion was supported by participants’ individual justifications, indicating that noticeable differences in the speed and pattern of eye movements were relevant to their judgements. As an important limitation of our study, we did not include a video condition with masked eyes. Such a condition would have told us to what extent temporal dynamics in expression (beyond the eye region) contribute to the recognition of attention states. Future research should also investigate the relevance of different eye parameters (i.e., eye position, eye movement) as well as eye-related facial cues (e.g., upper lid raiser, AU5; lid tightener, AU7) to better understand what specific physiological indicators within the eye region provide most critical information of external and internal mental states.

We also found that people engaged in demanding mental tasks, both internal and external, were more likely judged to be externally focused. This effect was observed in conditions with eyes masked and non-masked, indicating that this effect is associated with facial cues beyond the eye region. Higher cognitive effort may have resulted in more tensed facial expressions, which can easily be mistaken as higher visual engagement (Whitehill, Serpell, Lin, Foster, & Movellan, 2014). One implication of this is that undemanding internal cognition will be more easily recognized by others than states of intense deliberations. Moreover, complex mental imagery sometimes involves similar eye movements as would be observed during actual perception of the imagined scenes (e.g., Johansson, Holsanova, & Holmqvist, 2005). Together, these cues may give a false impression of external focus.

Overall, judgement accuracy was modest, even for dynamic facial displays. In part, this might be explained by the highly standardized experimental setting that aimed to remove all alternative sources of information beyond facial cues, potentially at the cost of external validity (Kingstone, 2009). Real-life face-to-face encounters involve much richer contextual information, which might increase attention recognition in everyday life (Achim, Guitton, Jackson, Boutin, & Monetta, 2013). For example, in teaching contexts we may observe the students response to presenting a new slide or to direct eye contact. Nonetheless, our findings show in controlled settings, that faces, and in particular the eyes, disclose whether attention is directed internally or externally.

These results bear important practical implications, e.g., for educational settings. The ability of lecturers and teachers to identify when they are losing their audience is critical as it allows them to undertake attempts to reengage their students. Moreover, if relevant facial cues can also be classified automatically, video recordings may serve to model someone’s external/internal attentional state, which could be used in automated tutoring systems (e.g., Putze, Küster, Annerer-Walcher, & Benedek, 2018; Whitehill et al., 2014) and potentially many other forms of computer-assisted activity (e.g., Smith, Shah, & da Vitoria Lobo, 2003).

## Data availability and deposition

De-identified performance data along with analysis scripts are available at Open Science Framework (https://osf.io/vga53; doi: 10.17605/OSF.IO/VGA53). Image and video stimuli have not been made available in order to protect the rights of the displayed persons.

## Notes

1 We additionally computed the hit rates for each experimental condition. In the video condition, the hit rates were 66.02%, 50.00%, 58.43%, and 73.70% for IDC with low and high demand and for EDC with low and high demand, respectively. In the image condition, the hit rates were 59.54%, 47.13%, 54.81%, and 61.67% for IDC with low and high demand and for EDC with low and high demand, respectively. In the masked image condition, the hit rates were 51.39%, 45.93%, 52.59%, and 54.26% for IDC with low and high demand and for EDC with low and high demand, respectively.

2 A three-way ANOVA considering the factors *attention*, *task demand*, and *presentation mode* revealed essentially the same results, but additionally showed various significant interactions with *presentation mode*. This is simply due to the fact that attention states were not recognized reliably in the masked image condition. Specifically, the three-way ANOVA showed significant main effects of *attention* (*F*[1,107] = 254.65; *p* < .001, $\eta_{p}^{2}$ = 0.70), *task demand* (*F*[1,107] = 149.15; *p* < .001, $\eta_{p}^{2}$ = 0.58), and *presentation mode* (*F*[2,214] = 4.81; *p* < .001, $\eta_{p}^{2}$ = 0.04). There was no significant interaction of *attention ** *task demand* (*F*[1,107] = 0.04; *p* = .84, $\eta_{p}^{2}$ = 0.00), but for *attention ** *presentation* mode (*F*[1.35,144.28] = 90.77; *p* < .001, $\eta_{p}^{2}$ = 0.46) and *task demand ** *presentation mode* (*F*[1.73,185.19] = 58.54; *p* < .001, $\eta_{p}^{2}$ = 0.35). We further observed a weak trend towards a significant interaction of *attention ** *task demand ** *presentation mode* (*F*[1.73,185.45] = 2.62; *p* = .08, $\eta_{p}^{2}$ = 0.02).
